# Challenges in Managing EBV-Associated T- and NK-Cell Lymphoproliferative Diseases

**DOI:** 10.3389/fped.2018.00320

**Published:** 2018-11-06

**Authors:** Yoshitaka Sato

**Affiliations:** Department of Virology, Nagoya University Graduate School of Medicine, Nagoya, Japan

**Keywords:** EBV, EBV-T/NK LPDs, basic-preclinical research, registry database, CAEBV

Epstein-Barr virus (EBV) infects >90% of adults worldwide and is closely linked to multiple B-cell malignancies, including Burkitt lymphoma, diffuse large B-cell lymphoma, Hodgkin lymphoma, and post-transplant lymphoproliferative disorder (PTLD) (1). Epstein-Barr virus also infects T-cells and natural killer (NK) cells causing EBV-associated T- and NK-cell (EBV-T/NK) malignancies, including extranodal NK/T-cell lymphomas, nasal type (ENKL), aggressive NK-cell leukemia, and lymphoproliferative diseases (LPDs). These EBV-associated T/NK-cell tumors have basically neoplastic properties with clonal proliferation and organ infiltration (2).

Chronic active EBV infection (CAEBV), an EBV-T/NK LPD, is a potential life-threatening illness in children and young adults, characterized by the clonal proliferation of EBV-infected lymphocytes (3, 4). The T/NK-cell type of this disease is more frequent in East Asians and some Native American populations in Western countries. CAEBV patients from the United States more often have EBV in B- or T-cells (3, 5). Patients with CAEBV often progress to overt lymphoma or leukemia. Although concurrent chemoradiotherapy along with non-anthracycline-based chemotherapy has improved the survival of patients with these EBV-T/NK malignancies, the survival outcome remains poor because of relapse or treatment-related mortality (6). The only curative treatment is stem-cell transplantation, albeit the incidence of transplantation-related complications is high (7, 8). To improve the treatment of EBV-T/NK malignancies, novel approaches using molecular targets have been attempted (Table 1).

**Table 1:** Recent findings of targeted therapies for EBV-T/NK LPDs.

**Class**	**Drug**	**Target**	**Note**	**References**
Monoclonal antibody	Pembrolizmab	PD-1	Five of 7 relapsed/refractory ENKL patients in at least clinical complete responses (CRs)	(9)
	Mogamulizumab	CCR4	Growth inhibition of EBV-associated T/NK-cell lymphoma in murine xenograft model	(10)
HDAC inhibitor	SAHA	HDAC	Tumor growth suppression in murine xenograft model	(11)
	Romidepsin	HDAC	Discontinued due to the EBV reactivation-associated adverse events	(12)
Hsp90 inhibitor	BIIB021	LMP1	Suppression of EBV-positive NK-cell growth in murine xenograft model	(13)
mTOR inhibitor	Rapamycin, CCI-779	Akt/mTOR pathway	Inhibition of EBV-associated T/NK-cell lymphoma growth in NOG mice	(14)
JAK inhibitor	Ruxolitinib	JAK1, JAK2	Suppression of inflammatory cytokines production in CAEBV patient-derived cells	(15)
	Tofacitinib	JAK3	Tumor growth inhibition in EBV-associated T-cell lymphoma in NOG mice	(16)
Proteasome inhibitor	Bortezomib	Ubiquitin-proteasome system	Suppression of EBV-associated tumor growth in murine xenograft model	(17, 18)

Immune checkpoint blockade with monoclonal antibodies directed at the inhibitory immune receptors, programmed death 1 (PD-1) and programmed death ligand 1 (PD-L1), has emerged as a successful treatment approach for patients with advanced cancers. Since EBV-infected lymphoma cells upregulate PD-L1 (19), these molecules are, therefore, the target of the antitumor effect. Pembrolizumab, the humanized anti-PD-1 monoclonal antibody, is effective for relapsed/refractory ENKL (9), suggesting that checkpoint inhibitors have a promising effect in the treatment of relapsed disease.

In addition to checkpoint inhibitors, some antibodies and inhibitors are also treated as potential molecular therapeutic targets in the developmental and preclinical stages. Kanazawa et al. showed that CC chemokine receptor 4 (CCR4) was expressed on most EBV-infected T/NK-cell lines and a humanized anti-CCR4 monoclonal antibody, mogamulizumab, inhibited the growth of EBV-positive NK-cell lymphomas in a murine xenograft model (10). Another challenge is targeting histone deacetylase (HDAC). The HDAC inhibitors, suberoylanilide hydroxamic acid (SAHA) and romidepsin, have been approved by the United States Food and Drug Administration and their efficacies in non-Hodgkin lymphoma, acute myeloid leukemia, cutaneous T-cell lymphoma, and relapsed and refractory peripheral T-cell lymphoma have been confirmed by clinical trials (20–22). SAHA suppressed tumor progression and metastasis in a murine xenograft model, although there were no significant differences observed between EBV- positive and EBV-negative cell lines (11). However, a pilot study using romidepsin for the treatment of relapsed/refractory ENKL patients in Korea was discontinued due to serious adverse events. As romidepsin treatment caused EBV reactivation, patients developed fever and elevated liver enzyme and bilirubin levels immediately after their first dose of romidepsin (12). These results suggest that the further accumulation of evidence in the preclinical stage is required for safer application of drug candidates in clinical trials.

The EBV-encoded latent membrane protein 1 (LMP1) is a major oncogene that activates the nuclear factor kappa B (NF-κB), c-Jun N-terminal kinase (JNK), and phosphatidylinositol 3-kinase (PI3K) signaling pathways, thereby, promoting the cell growth and inhibiting apoptosis (23). LMP1 is expressed in EBV-infected T/NK-cells. Screening a library of small-molecule inhibitors identified heat shock protein 90 (Hsp90) inhibitors as suppressors of LMP1 expression (24). In EBV-positive cells, the synthetic Hsp90 inhibitor BIIB021 suppressed the LMP1 expression and that of its downstream signaling proteins NF-κB, JNK, and Akt. The BIIB021 inhibited the growth of established EBV-positive NK-cells in NOD/Shi-*scid*/IL-2Rγ^null^ (NOG) mice (13). Moreover, constitutive PI3K/Akt/mTOR activation is critically involved in EBV-associated B-cell lymphoma (25, 26). Kawada et al. demonstrated that intraperitoneal treatment with an mTOR inhibitor significantly inhibited the growth of EBV-associated NK-cell lymphomas in a murine xenograft model and decreased the EBV load in peripheral blood, while T-cell lines were more sensitive to the mTOR inhibitors, but there were no significant differences between EBV-positive and EBV-negative cell lines (14). A series of studies of the JAK-STAT axis in EBV-T/NK LPDs provided new insight into its development. The STAT3 was activated in T/NK-cells in six of seven patients with CAEBV, promoting survival and cytokine production (15). Indeed, the selective JAK3 inhibitor, tofacitinib, significantly inhibited the growth of established tumors in NOG mice (16). We have already demonstrated the antitumor activity of the proteasome inhibitor bortezomib on EBV-associated lymphoma cells (17, 18). Therefore, combining these agents is a promising strategy to improve the treatment of EBV-T/NK lymphomas.

A fundamental question regarding the etiology of EBV-T/NK LPDs remains. The precise mechanism of T/NK-cell tumorigenesis remains to be elucidated because EBV-T/NK tumors are rare, and the generation and handling of EBV-positive T/NK cells are more difficult than with B-cells. To elucidate the genetic background related to these rare tumors, next-generation sequencing (NGS), including whole-genome sequencing and whole-exome sequencing, is a powerful, unbiased approach. Mutations of DDX3X, TP53, BCOR1, and STAT3 have been found in Chinese (27) and Japanese (28) patients with ENKL, although the mutation rates differed between these cohorts. Li et al. showed that genetic variation at *HLA-DPB1* is a strong contributor to extranodal NK/T-cell lymphoma (29). These findings highlight a pathogenic link between genetic variation and EBV-associated neoplastic proliferation. However, the possibility that specific EBV strains or variants have a higher tendency to develop T/NK-cell tumors cannot be eliminated now. Notably, Kimura's group also revealed that the EBV genome in CAEBV patients harbored frequent intragenic deletions (Dr. Kimura, personal communication). The genetic data generated from NGS-based approaches are required for their subsequent validation as definitively disease causing. Therefore, patient registries and biospecimen repositories are needed to accelerate bridging research from the developmental and preclinical stages to a clinical setting. In Japan, a nationwide registry of EBV-T/NK LPDs has been started (currently only Japanese, https://www.med.nagoya-u.ac.jp/virus/caebv/). We hope that this registry will grow and be linked to international registries to improve the efficacy and quality of the treatment of EBV-associated tumors.

As Abraham Lincoln, the 16th president of the United States, once said, “I will prepare and someday my chance will come.”

## Author contributions

The author confirms being the sole contributor of this work and has approved it for publication.

### Conflict of interest statement

The author declares that the research was conducted in the absence of any commercial or financial relationships that could be construed as a potential conflict of interest.
